# Association of specific microbiota taxa in the amniotic fluid at birth with severe acute and longer-term outcomes of very preterm infants: a prospective observational study

**DOI:** 10.1186/s12916-025-04259-9

**Published:** 2025-07-18

**Authors:** Birte Staude, Silvia Gschwendtner, Tina Frodermann, Frank Oehmke, Thomas Kohl, Susanne Walch, Michael Schloter, Harald Ehrhardt

**Affiliations:** 1 Department of General Pediatrics and Neonatology, Justus Liebig University and Universities of Giessen and Marburg Lung Center, Giessen, Germany; 2 Division of Neonatology and Pediatric Intensive Care Medicine, Department of Pediatrics and Adolescent Medicine, University Medical Center Ulm, Ulm, Germany; 3 Research Unit for Comparative Microbiome Analysis, Helmholtz Zentrum München, German Research Center for Environmental Health, Neuherberg, Germany; 4 Department of Gynecology and Obstetrics, Justus Liebig University of Giessen, Giessen, Germany; 5 German Center for Fetal Surgery and Minimally Invasive Therapy (DZFT), University of Mannheim (UMM), Mannheim, Germany

**Keywords:** Preterm infant, Amniotic fluid, 16S rRNA, Microbiome, Respiratory distress, Late-onset infection, Intraventricular haemorrhage, Bronchopulmonary dysplasia, Retinopathy of prematurity, Psychomotor outcome

## Abstract

**Background:**

Dysbiotic microbial colonization predisposes to severe outcomes of prematurity, including mortality and severe morbidities like necrotizing enterocolitis (NEC), late-onset infection (LOI) and bronchopulmonary dysplasia (BPD). Here, we studied the variations in the bacterial signatures in the amniotic fluid (AF) of very preterm deliveries < 32 weeks with severe acute and longer-term outcomes within a prospective cohort study.

**Methods:**

One hundred twenty-six AF samples were available for 16S rRNA gene metabarcoding to describe bacterial community structure and diversity in connection to intraventricular haemorrhage (IVH), LOI, focal intestinal perforation (FIP), NEC, retinopathy of prematurity (ROP) and the 2-year cognitive (MDI) and motor (PDI) outcome.

**Results:**

Diversity and overall bacterial community composition did not differ between the studied outcomes. But disparities in sequences assigned to single bacterial taxa were observed for the acute outcomes LOI and ROP and the longer-term impairments of MDI and PDI. Enrichments associated with a poor acute outcome were particularly detected in the *Escherichia-Shigella* cluster, while the predominance of *Ureaplasma* and *Enterococcus* species was associated with unrestricted acute and longer-term outcomes. Analysis for FIP did not reach any significance. IVH and NEC constituted rare events, prohibiting the analyses.

**Conclusions:**

Our data provide evidence that microbiota patterns at birth might allow the early identification of infants at risk for the severe outcomes of prematurity and argue against morbidity-specific associations. The data support the early origins hypothesis and relevant contribution of prenatal factors. The partly existing disparities between acute and longer-term outcomes might be traced back to the relevant impact of the diverse longitudinal exposures and socioeconomic factors.

**Supplementary Information:**

The online version contains supplementary material available at 10.1186/s12916-025-04259-9.

## Background

During recent years, it became more and more evident that physiologic microbial signatures are relevant for somatic and mental health across the lifespan [1, 2]. Microbial dysbiosis has been linked to major morbidities of all organs and to major and epidemiologically relevant acute and chronic diseases [3, 4]. Thereby, direct microbiota effects were determined for the lung and gastrointestinal tract, but remote actions via microbial axes were described for the endocrine function and the brain as well, and associations were observed across the complete life span [3–6]. For the newborn infant, aberrant microbial colonization at or after birth has been described as a major trigger for persistent changes in the microbiota structures and associated with chronic diseases later in life, including allergic diseases like asthma, autoimmunologic disorders, diabetes and neurodegenerative disorders [3–9]. While the observational studies in humans can only provide associations but were not able to prove causality, experimental models undoubtedly documented the patho-mechanistic link between dysbiotic microbiota structures and disease origin [10–13].

Very preterm (VPT) infants are at high risk for severe acute morbidities during their initial stay in the neonatal intensive care unit (NICU) including bronchopulmonary dysplasia (BPD), intraventricular haemorrhage (IVH), necrotizing enterocolitis (NEC), retinopathy of prematurity (ROP) and late onset infections (LOI), which have dramatic consequences for the overall acute health status and the long-term perspective [14–17]. Despite all the advances in medical care during their stay in the NICU, VPT infants are still at high risk for impairment and long-term sequelae, and the overall disease burden was not reduced for most of the outcomes during the last 25 years [18]. This unmet need was attributed to the prenatal origins of the diseases, which was best studied for BPD, where, for example, amniotic infection and preeclampsia/eclampsia/HELLP are drivers for prenatal disease development [12, 13]. For BPD, sophisticated newborn animal models have confirmed the role of dysbiotic microbiota structures and microbial toxins as disease drivers. These observations are likely also true for the other severe outcomes [13, 19]. Congruent insights were obtained in prospective observational cohort studies in preterm infants with analyses of their microbiota structures. The colonization of the respiratory tract directly after birth with bacterial species which have the potential to act as pathogens during the stay in the NICU was associated with a more severe pulmonary outcome [20–23]. The prenatal origins of BPD were recently confirmed on amniotic fluid samples obtained immediately before delivery. While the microbiota signatures differed vastly from that of intact pregnancies, they did not reveal overall disparities in the total microbial load or diversity between the different severity stages of BPD, except for partly increased evenness. But variations in amplicon sequence variants (ASV) assigned to the *Escherichia-Shigella* cluster and *Gardnerella*, *Enterococcus* and *Ureaplasma* species were associated with BPD disease severity [24]. Together, these data explain why postnatal improvements in medical care did not prevent BPD but were only suited to alleviate the postnatal aggravation of lung injury and severity stage of BPD [25, 26]. While best studied for the immature lung, for the infection-triggered diseases of LOI and NEC as well, convincing evidence exists that the clinical management, including antibiotic therapy and provision of breast milk, highly impacts the microbiota structures and risks [27, 28]. For the other severe outcomes of prematurity, like IVH or ROP, and the longer-term psychomotor outcome, such well-founded experimental insights or profound association studies are so far not available.

Here we evaluated the associations of bacterial signatures in the amniotic fluid at birth with the outcomes of VPT birth within a prospective cohort study. We assessed variations in diversity, overall community composition and ASV assigned to specific bacteria for the various acute severe outcomes and the cognitive and motor outcome at 2 years of age.

## Methods

### Study population and definition of baseline characteristics and outcomes

We executed our analyses on the recently described population of preterm infants < 32 weeks of gestation that were prospectively enrolled within the PROTECT-AIRR cohort study at the perinatal centre Giessen between July 2015 and May 2020. The study was conducted according to the rules of the Declaration of Helsinki, was approved by the ethics committee at the Justus-Liebig-University of Gießen (Az 135/12) and was registered at the DRKS (DRKS00004600). After provision of oral information, we obtained written informed consent from the parents of all preterm infants participating in the study.

The clinical data of patients included in the study were retrieved from the electronic data management system and the paper file records, as done before [29]. Baseline maternal and neonatal characteristics included gestational age at delivery, birth weight and z-score, gender, mode of delivery, cause for preterm delivery, birth as singleton or multiple and Apgar score. Small for gestational age (SGA) status was defined as birth weight < 10th percentile according to the nomograms of the German perinatal registry [30]. Prenatal antibiotic therapy was documented when applied within the last 7 days before delivery. Cause of preterm delivery for intra-amniotic infection was counted when at least one of the following criteria was fulfilled: documented histologic chorioamnionitis, an elevated amniotic fluid (AF) interleukin-6 > 3600pg/ml, the presence of intractable premature contractions during tocolytic therapy or the premature rupture of membranes before the onset of labour. The presence and severity of BPD were graded according to the NICHD consensus definition as applied recently. Infants who required supplemental oxygen for ≥ 28 days of life were categorized as mild BPD if they were on room air without any respiratory support at 36 weeks postmenstrual age. If infants required supplemental oxygen < 30% at 36 weeks, they were categorized as moderate BPD and with ≥ 30% of oxygen and/or need for positive pressure support as severe BPD [31]. High-flow nasal cannula therapy was accepted to provide continuous positive airway pressure with a PEEP equivalent of ≥ 3 cm H2O and calculation of the fraction of oxygen by low-flow nasal cannula was done as published recently [32, 33]. IVH severity was staged from the ultrasound examinations using the Deeg definition [34] and maximum stage of ROP from the screening examinations executed during clinical routine. LOI were counted when the criteria of the German NICU nosocomial surveillance system (NEO-KISS) were fulfilled [35]. Cognitive (MDI) and motor (PDI) outcomes at 24 months corrected age were evaluated with the Bayley III assessment battery. Clinically relevant cognitive and motor deficits were accepted with a score < 85 as published before [36, 37].

### Amniotic fluid sample collection

AF samples were obtained during routine caesarean delivery; for four cases, the collection was executed during amniotic puncture immediately before the decision to VPT delivery. As all AF samples were obtained by amniotic puncture or during caesarean section immediately before delivery, microbial contamination of samples during spontaneous delivery by the microbiota of the birth channel was excluded. AF samples were directly collected into sterile pyrogen-free protein low-bind tubes (Eppendorf SE, Hamburg, Germany) and immediately stored at − 80° until analysis.

### DNA extraction, 16S rRNA gene sequencing and data processing

Bacterial DNA extraction, 16S ribosomal RNA (16S rRNA) gene sequencing and data analyses were all executed as described recently [24]. Shortly, AF samples of 1.5mL in volume were centrifuged before pellet digestion with lysozyme (20 mg/ml) and Proteinase K (20 mg/ml) and DNA extraction with phenol–chloroform [38]. Subsequently, the V3–V4 hypervariable region of the 16S rRNA gene was amplified. Indexing PCR products were purified with AMPure XP beads and quantified on Fragment Analyzer™ (Advanced Analytical Technologies, Inc., Ankeny, USA) before sequencing on a MiSeq Illumina instrument (MiSeq Reagent Kit v3 (600 Cycle); Illumina, San Diego, CA, USA) [39]. For contamination surveillance, parallel blank extraction samples and PCR no-template controls were included (*n* = 3; for one, no PCR product could be obtained). Only FASTQ files with a minimum read length of 50 and a minimum Phred score of 15 were included in the final analysis performed with the QIIME 2 software package (version 2019.10.0) using the DADA2 plugin for quality control and SILVA_132_QIIME release 99% for taxonomic assignment [40, 41]. Blank samples showed clearly reduced richness and higher evenness as well as different bacterial community composition compared to AF samples (Additional File 1: Additional Figure S1). To exclude potential contamination, ASV occurring in extraction and PCR controls were removed from the dataset (33 and 2 ASV, respectively) (Additional File 2: Table S1). Additionally, mitochondrial sequences and singletons were removed, resulting in 1,558,601 reads in total, ranging from 69,785 to 114 reads per sample (median 12,771 reads per sample) (Additional File 2: Table S2).

Rarefaction curve analysis showed a sufficient sampling depth at 5000 reads (Additional file 1: Figure S2). Consequently, samples with reads < 5000 (*n* = 39) were excluded from subsequent analysis, and data normalization was done by subsampling to 5434 reads (the lowest obtained read number in remaining samples, *n* = 77). All sequence data of the study are deposited in the short read archive of NCBI and accession number PRJNA1260988.

### Quantitative real‑time PCR

Quantitative real-time PCR (qPCR) of the 16S rRNA gene as proxy for bacterial load was performed as described previously [24], using primers FP 16S/RP 16S and the following PCR conditions: 10 min at 95 °C; 40 cycles of 45 s at 95 °C, 45 s at 58 °C, 45 s at 72 °C; 10 min 72 °C; 1 cycle of 15 s at 95 °C, 30 s at 60 °C, 15 s at 95 °C. The quantified gene copy numbers were normalized to 1 ml of amniotic fluid. The bacterial load in blank extraction controls and PCR no template controls (*n* = 15) ranged from below detection level to one to three orders of magnitude lower compared to AF samples (Additional file 1: Additional Figure S1).

### Statistical analysis

All statistics were performed in R version 4.2.1 (https://www.R-project.org). Qualitative data were analysed with chi-square test and Fisher test as appropriate and with Benjamini–Hochberg adjustment for multiple comparisons. Bacterial loads quantified via qPCR were analysed using Kruskal–Wallis test and Wilcoxon-rank sum test, as appropriate. Alpha diversity was calculated using species richness based on ASV number, Pielou’s evenness and Shannon diversity index. Beta diversity was analysed via unweighted and weighted UniFrac distance. For statistical purposes, Kruskal–Wallis test, Wilcoxon-rank sum test and PERMANOVA with Benjamini–Hochberg *p* value correction for multiple comparisons were used. To identify microbial taxa differing between the analysed groups, a generalized linear model (R package MASS) was used. For model validation, residual histograms, plots showing sample quantiles versus theoretical quantiles and plots showing residuals versus fitted values were checked for normal distribution and variance homogeneity of residuals [42]. Significant differences (*p* < 0.05) were calculated via ANOVA respective Wilcoxon and Kruskal–Wallis test using Benjamini–Hochberg *p* value adjustment for multiple pairwise comparisons. Additionally, differential expression analysis via edgeR was performed to verify the model results. Only taxa with *p* < 0.05 in both methods were considered as significant. Plots were created in R using ggplot2, ggpubr and metacoder. Additionally, linear regression with the outcomes of richness as well as abundance of *Bifidobacteria*, *Enterococcus*, *Ureaplasma* and *Escherichia-Shigella*, respectively, was used to identify possible confounding by birth weight.

## Results

### Cohort characteristics

**Fig. 1:**
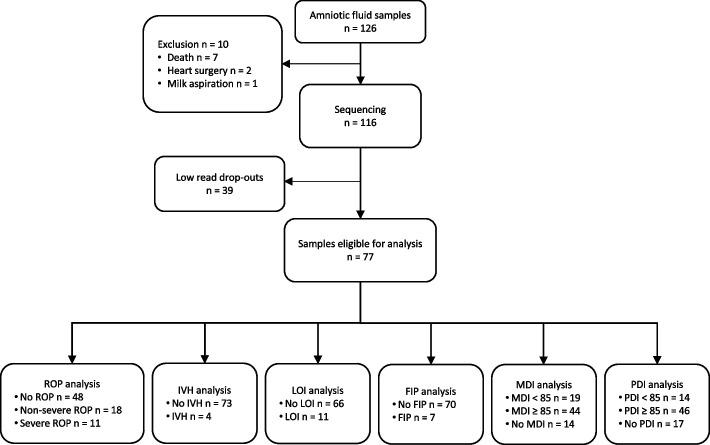
Study population flow chart. Flow chart of inclusion and exclusion of infants into the study population. Exclusion criteria included AF samples with low read bacterial signal, death before the outcome estimates and the severe morbidities of prematurity with high impact on the outcomes studied. ROP, retinopathy of prematurity; IVH, intraventricular haemorrhage; LOI, late onset infection (>72 hours after birth); FIP, focal intestinal perforation; MDI, mental developmental index; PDI, psychomotor developmental index

Clinical outcome data and amniotic fluid (AF) samples obtained immediately before delivery were available from overall 126 VPT infants. Seven patients were excluded from the analyses due to death before 36 weeks of gestation, where we were not able to determine the outcomes (Fig. 1). Two infants were excluded for heart surgery for congenital heart defects and one after complications due to milk aspiration. A further 39 infants were excluded as their AF samples rendered low bacterial reads [24, 43]. Infants with low bacterial reads did not differ in their baseline characteristics or outcomes compared to infants included in the analyses (Table 1). Overall, 77 infants were available for the acute outcomes studied. Follow-up data were available for MDI from 63 and for PDI from 60 infants (Fig. 1). Further infant characteristics are detailed specifically for each outcome analysed within the respective subsequent sections.

**Table 1:** Baseline characteristics and outcomes separated into infants included into the analysis and infants excluded for a low number of reads in the amniotic fluid samples

Exclusion for low reads			
	Included	Low read	*p* value
	*n* = 77	n = 39	
Baseline characteristics			
Weeks	28 (26,30)	27 (26,29)	0.69
Birth weight [g]	960 (750,1350)	950 (775,1145)	0.46
Small for gestational age	12 (15.6%)	9 (23.1%)	0.46
Female	34 (44.2%)	19 (48.7%)	0.79
Singleton	44 (57.1%)	21 (53.8%)	0.89
Caesarean section	74 (96.1%)	38 (97.4%)	1.00
Aetiology of preterm birth			0.36
AIS	34 (44.2%)	13 (33.3%)	
HELLP/preeclampsia	14 (18.2%)	12 (30.8%)	
IUGR	14 (18.2%)	5 (12.8%)	
Other	15 (19.5%)	9 (23.1%)	
ANCS			0.88
None/< 24 h	19 (24.7%)	10 (25.6%)	
24 h–7 days	39 (50.6%)	21 (53.8%)	
> 7 days	19 (24.7%)	8 (20.5%)	
Morbidities and outcomes			
BPD severity			0.71
No BPD	31 (40.3%)	13 (33.3%)	
Mild BPD	27 (35.1%)	14 (35.9%)	
Moderate/severe BPD	19 (24.7%)	12 (30.8%)	
ROP severity			0.36
No ROP	48 (62.3%)	20 (51.3%)	
Non-severe ROP	18 (23.4%)	14 (35.9%)	
Severe ROP	11 (14.3%)	5 (12.8%)	
IVH	4 (5.19%)	1 (2.56%)	0.66
Infection	11 (14.3%)	9 (23.1%)	0.36
FIP	7 (9.09%)	1 (2.56%)	0.26
NEC	0 (0.0%)	0 (0.0%)	
MDI < 85	19 (30.2%) (na = 14)	7 (30.4%) (na = 7)	1.00
PDI < 85	14 (23.3%) (na = 17)	6 (26.1%) (na = 6)	1.00

Qualitative data is presented as n with the proportion in brackets. Quantitative data is presented as median with 1st and 3rd quartiles in square brackets. For statistical analyses, Kruskal–Wallis and Pearson tests were used as appropriate

*Abbreviations*: *AIS* amnion infection syndrome, *IUGR* intrauterine growth restriction, *ANCS* antenatal corticosteroids, *BPD *bronchopulmonary dysplasia, *ROP* retinopathy of prematurity, *IVH* intraventricular haemorrhage, *ROP *retinopathy of prematurity, *FIP* focal intestinal perforation, *MDI* mental developmental index, *PDI* psychomotor developmental index

### Microbial signatures at birth and severe outcomes during the longitudinal stay in the NICU

**Fig. 2:**
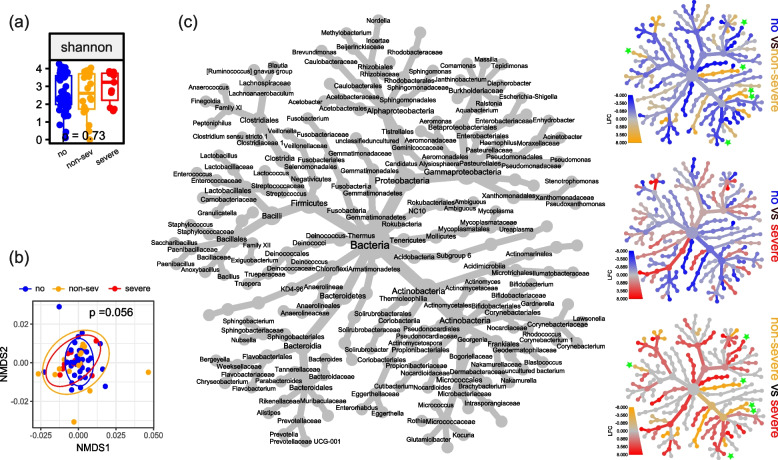
Differences in 16S rRNA gene microbial abundance in AF samples from preterm deliveries and ROP. **a **Alpha diversity measured as Shannon diversity index shows no significant (*p* < 0.05) difference between AF samples of preterm deliveries with no (blue), non-severe (orange) and severe (red) retinopathy of prematurity (ROP). Statistical analysis was performed using Wilcoxon Rank-Sum test. **b** NMDS plot of weighted Unifrac distances shows no significant (*p* < 0.05) altered bacterial 16S rRNA gene community composition for AF samples of preterm deliveries from Figure 2a. Statistical analysis was performed using PERMANOVA with Benjamini-Hochberg correction for multiple comparisons. **c** Heat tree of log-fold changes calculated with edgeR including top 100 genera (accounting for 97% of all reads in median). The labelled tree on the left shows the taxonomic information (domain to genus) and is the key for the unlabelled smaller trees. Smaller trees represent a comparison between no, non-severe and severe ROP. Coloured taxa are more abundant in the samples indicated by the coloured subtitle. Significant changes (p < 0.05 in both edgeR and generalized linear model) are marked with green asterisks

**Fig. 3:**
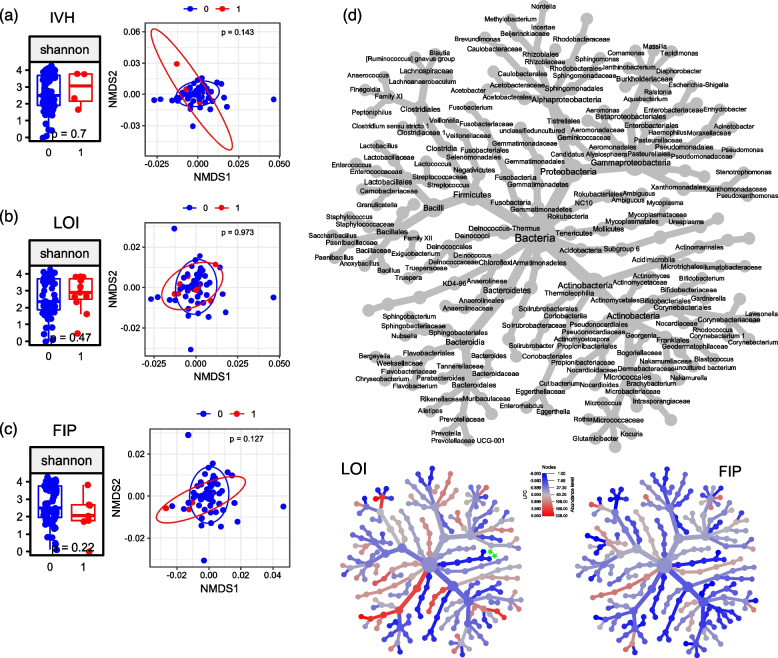
Differences in 16S rRNA gene microbial abundance in AF samples from preterm deliveries and IVH/LOI/FIP. **a** Alpha diversity (Shannon index) index and beta diversity (NMDS plot of weighted Unifrac distances) shows no significant (p < 0.05) difference in AF samples of preterm deliveries with (red) and without (blue) intraventricular haemorrhage (IVH). Statistical analysis was performed using Wilcoxon Rank-Sum test and PERMANOVA with Benjamini-Hochberg correction for multiple comparisons, respectively. **b** Alpha diversity (Shannon index) index and beta diversity (NMDS plot of weighted Unifrac distances) shows no significant (*p* < 0.05) difference in AF samples of preterm deliveries with (red) and without (blue) late onset infection (LOI). Statistical analysis was performed using Wilcoxon Rank-Sum test and PERMANOVA with Benjamini-Hochberg correction for multiple comparisons, respectively. **c** Alpha diversity (Shannon index) index and beta diversity (NMDS plot of weighted Unifrac distances) shows no significant (*p* < 0.05) difference in AF samples of preterm deliveries with (red) and without (blue) focal intestinal perforation (FIP). Statistical analysis was performed using Wilcoxon Rank-Sum test and PERMANOVA with Benjamini-Hochberg correction for multiple comparisons, respectively. **d** Heat tree of log-fold changes calculated with edgeR including top 100 genera (accounting for 97% of all reads in median). The labelled upper tree shows the taxonomic information (domain to genus) and is the key for the unlabelled smaller trees. Smaller trees represent a comparison between the patient groups from Figure 4b and 4c, respectively (blue: increased in 0, red: increased in 1) . Significant changes (*p* < 0.05 in both edgeR and generalized linear model) are marked with green asterisks

First, we evaluated the bacterial 16S rRNA gene signatures in the AF of infants with further acute severe outcomes of prematurity occurring during the longitudinal course in the NICU. For ROP, we segregated infants by the maximum disease severity into no ROP (*n* = 48), non-severe ROP (*n* = 18) and severe ROP (*n* = 11) where the last category included all stage 3 cases or higher at high risk for impaired visual acuity irrespective of the indication for ROP therapy or not. Infants with severe ROP had a lower gestational age (median 25 vs 28 weeks) and birth weight (median 590 g vs 1230 g) at birth; further baseline characteristics and outcomes are detailed in Table 2. Whereas bacterial load was significantly higher in the non-severe group, alpha and beta diversity did not differ between AF samples of infants for the three ROP categories (Fig. 2a and b, Additional File 1: Additional Figure S3a). But differences on single genus level were observed: When AF samples of infants with severe ROP were compared to the non-severe ROP category, ASV assigned to the *Escherichia*-*Shigella* group and *Kocuria* were more abundant in AF samples of infants with severe ROP while *Ureaplasma*, *Bifidobacterium, Gardnerella and Enterococcus* species displayed higher abundance in AF samples of infants with non-severe ROP (Fig. 2c). For IVH and LOI, FIP and NEC, total bacterial loads, alpha and beta diversity were analysed using the identical approach, and no statistically significant differences were observed. AF samples of infants who developed LOI had a lower abundance of ASV assigned to *Ureaplsma and Mycoplasma*. No significant differences could be observed for FIP. IVH constituted a rare event (four cases) in our cohort, prohibiting reliable in-depth statistical analysis due to highly unbalanced data. Consequently, analysis on single genera was not performed (Fig. 3, Additional File 1: Additional Fig. 3b-d, Tables 3, 4 and 5). Analyses for NEC were not performed, as there was only one case with NEC in our cohort. As birth weight prevailed as the only patient characteristic that displayed significant differences throughout the comparisons of infants with and without a severe morbidity, we additionally executed linear regression analyses. Throughout the analyses, no significant associations were detected between birth weight and bacterial richness as well as the species of interest, excluding birth weight as a possible confounder (Additional File 2: Supplemental Table S3).

**Table 2:** Baseline characteristics of infants separated by the severity of retinopathy of prematurity

ROP				
	No ROP	Non-severe	Severe	*p* value
	*n* = 48	*n* = 18	*n* = 11	
Baseline characteristics				
Weeks	29 (28,30)	26 (25,27)	25 (24,25)	< 0.001
Birth weight [g]	1230 (948,1452)	812 (668,938)	590 (535,695)	< 0.001
Small for gestational age	5 (10.4%)	2 (11.1%)	5 (45.5%)	0.021
Female	21 (43.8%)	8 (44.4%)	5 (45.5%)	0.99
Singleton	23 (47.9%)	15 (83.3%)	6 (54.5%)	0.034
Caesarean section	46 (95.8%)	17 (94.4%)	11 (100%)	0.75
Aetiology of preterm birth				0.17
AIS	18 (37.5%)	13 (72.2%)	3 (27.3%)	
HELLP/preeclampsia	9 (18.8%)	3 (16.7%)	2 (18.2%)	
IUGR	10 (20.8%)	1 (5.56%)	3 (27.3%)	
Other	11 (22.9%)	1 (5.56%)	3 (27.3%)	
ANCS				0.14
None/< 24 h	13 (27.1%)	4 (22.2%)	2 (18.2%)	
24 h–7 days	20 (41.7%)	10 (55.6%)	9 (81.8%)	
> 7 days	15 (31.2%)	4 (22.2%)	0 (0%)	
Morbidities and outcomes				
BPD severity				< 0.001
No BPD	28 (58.3%)	3 (16.7%)	0 (0%)	
Mild BPD	16 (33.3%)	10 (55.6%)	1 (9.09%)	
Moderate/severe BPD	4 (8.33%)	5 (27.8%)	10 (90.9%)	
IVH	2 (4.17%)	1 (5.56%)	1 (9.09%)	0.77
Infection	1 (2.08%)	4 (22.2%)	6 (54.5%)	< 0.001
FIP	2 (4.17%)	1 (5.56%)	4 (36.4%)	0.012
MDI < 85	8 (21.6%) (na = 11)	5 (31.2%) (na = 2)	6 (60.0%) (na = 1)	0.075
PDI < 85	4 (11.4%) (na = 13)	4 (23.5%) (na = 1)	6 (75.0%) (na = 3)	0.001

Qualitative data is presented as n with proportion in brackets. Quantitative data is presented as median with 1^st^and 3^rd^ quartile in square brackets. For statistical analyses Kruskal–Wallis and Pearson tests were used as appropriate

*Abbreviations*: *AIS* amnion infection syndrome, *IUGR* intrauterine growth restriction, *ANCS* antenatal corticosteroids, *BPD *bronchopulmonary dysplasia, *ROP* retinopathy of prematurity, *IVH* intraventricular haemorrhage, *ROP *retinopathy of prematurity, *FIP *focal intestinal perforation, *MDI* mental developmental index, *PDI* psychomotor developmental index

**Table 3:** Baseline characteristics of infants with and without intraventricular haemorrhage

IVH			
	No IVH	IVH	*p* value
	*n* = 73	*n* = 4	
Baseline characteristics			
Weeks	28 (26,30)	26 (25,28)	0.26
Birth weight [g]	970 (770,1350)	788 (652,1089)	0.46
Small for gestational age	11 (15.1%)	1 (25.0%)	0.50
Female	33 (45.2%)	1 (25.0%)	0.63
Singleton	43 (58.9%)	1 (25.0%)	0.31
Caesarean section	70 (95.9%)	4 (100%)	1.00
Aetiology of preterm birth			0.48
AIS	32 (43.8%)	2 (50.0%)	
HELLP/preeclampsia	14 (19.2%)	0 (0%)	
IUGR	14 (19.2%)	0 (0%)	
Other	13 (17.8%)	2 (50%)	
ANCS			1.00
None/< 24 h	18 (24.7%)	1 (25.0%)	
24 h–7 days	37 (50.7%)	2 (50.0%)	
> 7 days	18 (24.7%)	1 (25.0%)	
Morbidities and outcomes			
BPD severity			0.55
No BPD	30 (41.1%)	1 (25.0%)	
Mild BPD	26 (35.6%)	1 (25.0%)	
Moderate/severe BPD	17 (23.3%)	2 (50.0%)	
ROP severity			0.77
No ROP	46 (63%)	2 (50.0%)	
Non-severe ROP	17 (23.3%)	1 (25.0%)	
Severe ROP	10 (13.7%)	1 (25.0%)	
Infection	10 (13.7%)	1 (25.0%)	0.47
FIP	7 (9.59%)	0 (0%)	1.00
MDI < 85	17 (28.3%) (na = 13)	2 (66.7%) (na = 1)	0.21
PDI < 85	14 (24.1%) (na = 15)	0 (0%) (na = 2)	1.00

Qualitative data is presented as n with proportion in brackets. Quantitative data is presented as median with 1st and 3rd quartiles in square brackets. For statistical analyses, Kruskal–Wallis and Pearson tests were used as appropriate

*Abbreviations*: *AIS* amnion infection syndrome, *IUGR* intrauterine growth restriction, *ANCS* antenatal corticosteroids, *BPD *bronchopulmonary dysplasia, *ROP* retinopathy of prematurity, *IVH* intraventricular haemorrhage, *ROP *retinopathy of prematurity, *FIP* focal intestinal perforation, *MDI* mental developmental index, *PDI* psychomotor developmental index

**Table 4:** Baseline characteristics of infants with and without late-onset infection

Infection			
	No infection	Infection	*p* value
	*n* = 66	*n* = 11	
Baseline characteristics			
Weeks	28 (27,30)	25 (24,25)	< 0.001
Birth weight [g]	982 (855,1390)	570 (540,675)	< 0.001
Small for gestational age	8 (12.1%)	4 (36.4%)	0.06
Female	27 (40.9%)	7 (63.6%)	0.28
Singleton	35 (53%)	9 (81.8%)	0.15
Caesarean section	63 (95.5%)	11 (100%)	1.00
Aetiology of preterm birth			0.96
AIS	28 (42.4%)	6 (54.5%)	
HELLP/preeclampsia	12 (18.2%)	2 (18.2%)	
IUGR	13 (19.7%)	1 (9.09%)	
Other	13 (19.7%)	2 (18.2%)	
ANCS			0.12
None/< 24 h	16 (24.2%)	3 (27.3%)	
24 h–7 days	31 (47%)	8 (72.7%)	
7 days	19 (28.8%)	0 (0%)	
Morbidities and outcomes			
BPD severity			< 0.001
No BPD	31 (47%)	0 (0%)	
Mild BPD	25 (37.9%)	2 (18.2%)	
Moderate/severe BPD	10 (15.2%)	9 (81.8%)	
ROP severity			< 0.001
No ROP	47 (71.2%)	1 (9.09%)	
Non-severe ROP	14 (21.2%)	4 (36.4%)	
Severe ROP	5 (7.58%)	6 (54.5%)	
IVH	3 (4.55%)	1 (9.09%)	0.47
FIP	4 (6.06%)	3 (27.3%)	0.06
MDI < 85	13 (24.5%) (na = 13)	6 (60.0%) (na = 1)	0.06
PDI < 85	9 (18.0%) (na = 16)	5 (50.0%) (na = 1)	0.08

Qualitative data is presented as n with the proportion in brackets. Quantitative data is presented as median with 1st and 3rd quartiles in square brackets. For statistical analyses, Kruskal–Wallis and Pearson tests were used as appropriate

*Abbreviations*: *AIS* amnion infection syndrome, *IUGR* intrauterine growth restriction, *ANCS* antenatal corticosteroids, *BPD *bronchopulmonary dysplasia, *ROP* retinopathy of prematurity, *IVH* intraventricular haemorrhage, *ROP *retinopathy of prematurity, *FIP* focal intestinal perforation, *MDI* mental developmental index, *PDI* psychomotor developmental index

**Table 5:** Baseline characteristics of infants with and without focal intestinal perforation

FIP			
	No FIP	FIP	*p* value
	*n* = 70	*n* = 7	
Baseline characteristics			
Weeks	28 (27,30)	25 (24.5,27)	0.015
Birth weight [g]	970 (805,1358)	590 (555,895)	0.047
Small for gestational age	9 (12.9%)	3 (42.9%)	0.072
Female	32 (45.7%)	2 (28.6%)	0.45
Singleton	41 (58.6%)	3 (42.9%)	0.45
Caesarean section	67 (95.7%)	7 (100%)	1.00
Aetiology of preterm birth			0.89
AIS	31 (44.3%)	3 (42.9%)	
HELLP/preeclampsia	13 (18.6%)	1 (14.3%)	
IUGR	12 (17.1%)	2 (28.6%)	
Other	14 (20%)	1 (14.3%)	
ANCS			0.67
None/< 24 h	18 (25.7%)	1 (14.3%)	
24 h–7 days	34 (48.6%)	5 (71.4%)	
> 7 days	18 (25.7%)	1 (14.3%)	
Morbidities and outcomes			
BPD severity			0.11
No BPD	30 (42.9%)	1 (14.3%)	
Mild BPD	25 (35.7%)	2 (28.6%)	
Moderate/severe BPD	15 (21.4%)	4 (57.1%)	
ROP severity			0.012
No ROP	46 (65.7%)	2 (28.6%)	
Non-severe ROP	17 (24.3%)	1 (14.3%)	
Severe ROP	7 (10%)	4 (57.1%)	
IVH	4 (5.71%)	0 (0%)	1.00
Infection	8 (11.4%)	3 (42.9%)	0.056
MDI < 85	17 (29.3%) (na = 12)	2 (40.0%) (na = 2)	0.63
PDI < 85	11 (20.0%) (na = 15)	3 (60.0%) (na = 2)	0.780

Qualitative data is presented as n with proportion in brackets. Quantitative data is presented as median with 1st and 3rd quartiles in square brackets. For statistical analyses Kruskal–Wallis and Pearson tests were used as appropriate

*Abbreviations*: *AIS* amnion infection syndrome, *IUGR* intrauterine growth restriction, *ANCS* antenatal corticosteroids, *BPD *bronchopulmonary dysplasia, *ROP* retinopathy of prematurity, *IVH* intraventricular haemorrhage, *ROP *retinopathy of prematurity, *FIP* focal intestinal perforation, *MDI* mental developmental index, *PDI* psychomotor developmental index

### Species-level microbiota variations and psychomotor outcome at 2 years of age

**Fig. 4:**
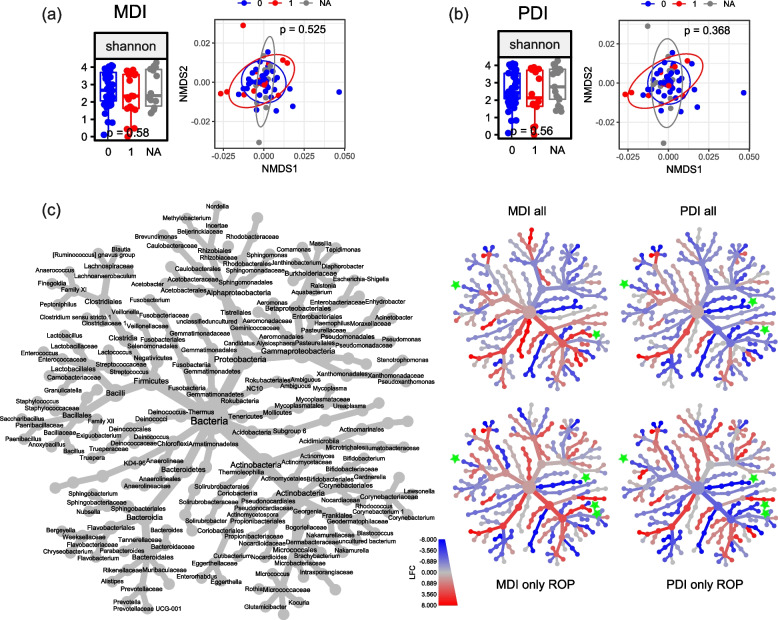
Differences in 16S rRNA gene microbial abundance in AF samples from preterm deliveries and neurological development. **a** Alpha diversity (Shannon index) and beta diversity (NMDS plot of weighted Unifrac distances) shows no significant (p < 0.05) difference in AF samples of preterm deliveries with mental developmental index (MDI) ≥85 (0, blue) and <85 (1, red). Statistical analysis was performed using Wilcoxon Rank-Sum test and PERMANOVA with Benjamini-Hochberg correction for multiple comparisons, respectively. **b** Alpha diversity (Shannon index) and beta diversity (NMDS plot of weighted Unifrac distances) shows no significant (*p* < 0.05) difference in AF samples of preterm deliveries with psychomotor developmental index (PDI) ≥85 (0, blue) and <85 (1, red). Statistical analysis was performed using Wilcoxon Rank-Sum test and PERMANOVA with Benjamini-Hochberg correction for multiple comparisons, respectively. **c** Heat tree of log-fold changes calculated with edgeR including top 100 genera (accounting for 97% of all reads in median). The labelled tree on the left shows the taxonomic information (domain to genus) and is the key for the unlabelled smaller trees. Smaller trees represent a comparison between the patient groups from Figure 4a and 4b, respectively (blue: increased in patients with index ≥85, red: increased in patients with index <85). The upper coloured trees include all patients (without those with missing outcome), the lower coloured trees only patients with ROP, left trees results for the MDI and right trees those for the PDI. Significant changes (*p* < 0.05 in both edgeR and generalized linear model) are marked with green asterisks

Lastly, we evaluated the association of specific microbial 16S rRNA gene signatures in the AF at birth with the cognitive and motor outcome at 2 years of age using the Bayley III Scales of Infant Development. Infants with an MDI < 85 were of lower gestational age (median: 25 vs 28 weeks) and were more likely to also have a PDI < 85 (Table 6). Infants with a PDI < 85 were significantly more immature (median 25 vs 28 weeks) and had a lower birth weight (median 590 g vs 970 g) had more BPD and severe ROP (Table 7). No differences in total bacterial loads, alpha and beta diversity in AF samples were observed between infants with an MDI or PDI ≥ 85 versus < 85 (Fig. 4a and b, Additional File 1: Figure S3e and S3f). But on the single genus level, enrichment in *Enterococcus* species in AF samples was associated with a normal outcome for both the MDI and PDI, while for *Ureaplasma* species, this was given only for PDI (Fig. 4c). For the *Escherichia-Shigella* cluster, no significant differences were detected even when the sample was restricted to those infants at highest risk for psychomotor impairment with a diagnosis of ROP, while the presence of *Enterococcus* and *Ureaplasma* prevailed highly significantly with an unrestricted psychomotor outcome (Fig. 4c).

**Table 6:** Baseline characteristics of infants with and without mental deficits

MDI			
	MDI ≥ 85	MDI < 85	*p* value
	*n* = 44	*n* = 19	
Baseline characteristics			
Weeks	28 (27,30)	27 (25,29)	0.03
Birth weight [g]	960 (792,1240)	870 (560,1200)	0.11
Small for gestational age	6 (13.6%)	4 (21.1%)	0.47
Female	22 (50%)	8 (42.1%)	0.76
Singleton	27 (61.4%)	12 (63.2%)	1.00
Caesarean section	42 (95.5%)	19 (100%)	0.87
Aetiology of preterm birth			0.23
AIS	16 (36.4%)	10 (52.6%)	
HELLP/preeclampsia	12 (27.3%)	1 (5.26%)	
IUGR	9 (20.5%)	4 (21.1%)	
Other	7 (15.9%)	4 (21.1%)	
ANCS			0.43
None/< 24 h	11 (25%)	6 (31.6%)	
24 h–7 days	26 (59.1%)	8 (42.1%)	
> 7 days	7 (15.9%)	5 (26.3%)	
Morbidities and outcomes			
BPD severity			0.06
No BPD	17 (38.6%)	6 (31.6%)	
Mild BPD	19 (43.2%)	4 (21.1%)	
Moderate/severe BPD	8 (18.2%)	9 (47.4%)	
ROP severity			0.08
No ROP	29 (65.9%)	8 (42.1%)	
Non-severe ROP	11 (25%)	5 (26.3%)	
Severe ROP	4 (9.09%)	6 (31.6%)	
IVH	1 (2.27%)	2 (10.5%)	0.21
Infection	4 (9.09%)	6 (31.6%)	0.05
FIP	3 (6.82%)	2 (10.5%)	0.63
PDI < 85	3 (7.14%) (na = 2)	10 (58.8%) (na = 2)	< 0.001

Qualitative data is presented as n with the proportion in brackets. Quantitative data is presented as median with 1st and 3rd quartiles in square brackets. For statistical analyses Kruskal–Wallis and Pearson tests were used as appropriate

*Abbreviations*:* AIS* amnion infection syndrome, *IUGR* intrauterine growth restriction, *ANCS* antenatal corticosteroids, *BPD* bronchopulmonary dysplasia, *ROP* retinopathy of prematurity, *IVH* intraventricular haemorrhage, *ROP* retinopathy of prematurity, *FIP* focal intestinal perforation, *MDI* mental developmental index, *PDI* psychomotor developmental index

**Table 7:** Baseline characteristics of infants with and without psychomotor developmental deficits

PDI			
	PDI ≥ 85	PDI < 85	*p* value
	*n* = 46	*n* = 14	
Baseline characteristics			
Weeks	28 (27,29.8)	25.5 (24.2,27)	0.0037
Birth weight [g]	970 (839,1240)	590 (555,838)	0.0035
Small for gestational age	5 (10.9%)	5 (35.7%)	0.08
Female	21 (45.7%)	8 (57.1%)	0.65
Singleton	27 (58.7%)	11 (78.6%)	0.30
Caesarean section	44 (95.7%)	14 (100%)	1.00
Aetiology of preterm birth			0.45
AIS	19 (41.3%)	8 (57.1%)	
HELLP/preeclampsia	10 (21.7%)	1 (7.14%)	
IUGR	9 (19.6%)	4 (28.6%)	
Other	8 (17.4%)	1 (7.14%)	
ANCS			0.077
None/< 24 h	11 (23.9%)	6 (42.9%)	
24 h–7 days	24 (52.2%)	8 (57.1%)	
> 7 days	11 (23.9%)	0 (0%)	
Morbidities and outcomes			
BPD severity			0.027
No BPD	19 (41.3%)	3 (21.4%)	
Mild BPD	19 (41.3%)	3 (21.4%)	
Moderate/severe BPD	8 (17.4%)	8 (57.1%)	
ROP severity			0.001
No ROP	31 (67.4%)	4 (28.6%)	
Non-severe ROP	13 (28.3%)	4 (28.6%)	
Severe ROP	2 (4.35%)	6 (42.9%)	
IVH	2 (4.35%)	0 (0%)	1.00
Infection	5 (10.9%)	5 (35.7%)	0.076
FIP	2 (4.35%)	3 (21.4%)	0.078
MDI < 85	7 (15.2%)	10 (76.9%) (na = 1)	< 0.001

Qualitative data is presented as n with the proportion in brackets. Quantitative data is presented as median with 1st and 3rd quartiles in square brackets. For statistical analyses, Kruskal–Wallis and Pearson tests were used as appropriate

*Abbreviations*: *AIS* amnion infection syndrome, *IUGR* intrauterine growth restriction, *ANCS* antenatal corticosteroids, *BPD *bronchopulmonary dysplasia, *ROP* retinopathy of prematurity, *IVH* intraventricular haemorrhage, *ROP *retinopathy of prematurity, *FIP* focal intestinal perforation, *MDI* mental developmental index, *PDI* psychomotor developmental index

## Discussion

Our data indicate that 16S rRNA gene signatures in the AF immediately before delivery differ between VPT infants, dependent on their acute and longer-term severe outcomes. While we recently described an association with the pulmonary outcome and the severity stages of BPD, we now provide evidence that comparable connections apply to further morbidities of prematurity [24]. This is not surprising as the important contribution of microbiota to health and disease was confirmed across the different organ systems and many disease entities [1–4]. Furthermore, the prominent role of bacterial infections in these morbidities was shown in the preclinical models and association studies in preterm infants [13, 19, 27, 44, 45]. The results of our study expand the association to the colonization at birth, as both infants with and without infections were at a higher risk for acute severe outcomes when specific bacterial strains were detected in their amniotic fluid at birth. While we described associations between the postnatal colonization with strains with potential pathogenicity and risk of BPD before, we now expand this association to the situation before preterm delivery [21, 24]. The importance of the data arises from the fact that it might provide insights into why postnatal therapies to reduce the risk of BPD, ROP or psychomotor impairment are only of limited efficacy when the initial injurious event occurred already before birth.

### Disparity in associations for the acute and longer-term morbidities

We did not detect an association of the Escherichia–Shigella cluster with the longer-term cognitive and motor impairment at 2 years of age. This might be surprising at first sight, but it has been well-described that the multiple longitudinal exposures during the stay in the NICU, variations in follow-up care and the socio-economic status of the family have a high impact that might be more relevant than the perinatal risks [29, 46, 47]. In line with our actual results, no associations between a low 5-min Apgar score and the 5-year psychomotor outcome were detected despite the close association with the acute severe outcomes [48–50]. Our results are further substantiated by a recent analysis on the missing association of chorioamnionitis and the 5-year psychomotor outcome [51].

### Putting the results into the context of the published literature

We were able to ascribe the associations to ASV assigned to specific bacterial genera with all severe outcomes in this and our previous analysis when the frequency of events allowed a valid statistical approach, while the overall diversity and community composition remained unchanged [24]. Most importantly and uniformly within the analyses, members of *Escherichia–Shigella* prevailed as the most relevant cluster associated with all acute severe outcomes, while enrichment in *Enterococcus* and *Ureaplasma* species complex mostly prevailed associated with better acute and unrestricted psychomotor outcomes. This is not surprising when considering the high frequency of detection of bacterial strains from the *Escherichia–Shigella* complex in connection with all acute severe morbidities in the clinics and the convincing experimental evidence that these strains with their LPS production induce signalling pathway alterations including activation of inflammation that result in injuries of the immature lung, eye and brain, which is comparable to the better studied and highly relevant postnatal injury of hyperoxia exposure [12, 13, 19, 29, 52–54]. In that direction, it was demonstrated before that microbiota structure dysbiosis drives innate immune responses and metabolomic pathologies that explain the prenatal damage to the immature organs of the preterm infant [23, 55]. While we observed mostly congruent results for the *Escherichia–Shigella* cluster throughout the analyses on the acute outcomes, there were disparities in the results for *Enterococcus* species [24]. This might reflect that some of the outcomes get differentially impacted by specific microbiota structures at birth, in addition to the larger differential impact on the longer-term outcomes, opening another dimension of diversity.

### Appeal for studies of varying pathogenicity on the single bacterial strain level

Due to the sample size, we were not able to provide association studies for further bacterial signatures with high pathogenicity, as their frequency of detection was too low in our cohort, in line with other studies on this topic [24, 43]. We were not able to address the interaction of different bacterial strains as described in sophisticated animal studies, for example, for preceding *Ureaplasma* species colonization of the amniotic cavity before the exposure to LPS from *Escherichia coli* that restricted the pro-inflammatory response following LPS by inducing an immunotolerance phenotype [56, 57]. Vice versa, those bacterial genera without the potential to induce pathogenicity and which have been described as important for the development of the infants like *Bifidobacterium species* were associated with a better outcome in our study, except for non-severe ROP compared to no ROP where we cannot provide a solid explanation. From this, it is imperative to expand the actual analyses beyond genus to the single strain to specify particularly harmful clusters within adequately powered prospective cohort studies.

### Strengths and limitations of the study

Our study relied on unique AF samples collected immediately before delivery by caesarean section under optimized conditions, excluding the contamination of the samples by the microbial spectrum and bacterial load of the maternal birth channel. Furthermore, we applied 16S rRNA gene-based sequencing to detect even low levels of bacterial genes, which is a well-described limitation of studies on this topic [24, 43]. Further advances are that we did not have to rely on bacterial cultivation techniques that would have been hampered by the prenatal antibiotic treatment of the pregnancies with impending delivery or application immediately before delivery by caesarean section during clinical routine. But our study has limitations as well. There were some differences in baseline characteristics between the infants with and without severe outcomes. Due to the limited sample size, we were not able to apply, for example, propensity score matching to account for these disparities. But regression analyses did not detect interactions by birth weight as the most relevant confounder. And we recently showed for BPD on this cohort and now for ROP and the 2-year psychomotor outcome that when comparing disease severity stages, the disparity in baseline characteristics was no more pronounced, arguing for bacterial strain-specific effects and not changes in colonization during different gestational ages [24]. We excluded a systematic bias by sample selection as samples from patients included and excluded for low bacterial reads did not differ in their baseline characteristics. Furthermore, the sample volumes were too small to expand the studies to long-read sequencing to specify the pathogenicity of the specific bacterial strains. The number of samples was limited due to the single-centre approach and the restricted time interval of sample collection. For this, further categorization beyond the morbidity level and to respect, for example, sex specific variations and the impact of variation in hygiene measures was not feasible [58, 59]. But as no changes in clinical treatment relevant to the study outcomes were introduced during the sample collection period and the frequencies of the severe outcomes did not change over time (data not shown), we can exclude the introduction of a systematic bias to the results. We cannot completely exclude skin bacterial contamination during caesarean deliveries, but samples were first obtained after opening of the uterus and skin commensals account for an average of 11% of reads in the samples. Furthermore, it remains a controversial discussion whether the AF harbours a vivid microbiome [60, 61]. It was not possible for us to verify bacterial viability, as perinatal antibiotic therapy during caesarean section is standard of care according to the guidelines in Germany. But the studies on *Ureaplasma* colonization in pregnancies with an intact amniotic membrane clearly indicate that the AF is not a sterile environment and that the rupture of membranes is not a prerequisite for bacterial invasion [62, 63]. Sophisticated animal studies might help to prove the invasion of a vivid microbiome and the transmission of bacteria from other sites like the gut, oropharynx and further body niches as postulated in the available literature on this topic [64–66]. The available literature on microbiota structures determined by 16 s rRNA gene sequencing indicates changing risks with microbial dysbiosis [67–69]. Our findings of microbiota structures in the AF of infants born preterm are in line with a recently published study that did find microbiota structures in pregnancies with an adverse outcome as well. Overall, the two studies give important insights into the microbiota dynamics in the amniotic fluid before and after the onset of labour [70]. However, even if there is no vivid intra-amniotic microbiome, the detected bacterial 16S rRNA genes clearly indicate an association to the acute severe outcomes and the discrimination might be of relevance when considering that bacterial toxins like LPS are the primary executors of organ injury not only in the preterm infant [12, 13, 71]. Lastly, confirmation via qPCR absolute copy numbers would have been advantageous [70]. Due to the small volumes of these unique samples, no material was left after quantification of bacterial load via 16S rRNA gene-based qPCR and sequencing; thus we were not able to do this additional analysis.

## Conclusions

The presented analyses specify the impact of the microbiota structures in the AF at birth on most of the acute severe outcomes of VPT birth and expand the associations far beyond the lung, while longer-term morbidities remained unchanged. The results are in line with previous observations that infants with BPD more frequently have a more severe respiratory course after birth and more frequently experience severe ROP [52, 72–75]. While the scientific question is open whether severe morbidities like BPD predispose or cause further severe outcomes, the data from our actual analysis indicate that the acute and longer-term severe outcomes have at least overlapping, if not common, early origins of disease origination [75–77]. And they indicate that the early origins are not based on the immaturity per se and that early disease drivers exist even before preterm delivery that have a relevant impact on the outcomes and severity of the morbidities in the preterm infant [12, 13, 74, 77]. This opens an important aspect to direct future therapeutic strategies intended to prevent acute severe outcomes. It might be too late to intervene during the longitudinal course in the NICU or even immediately after birth, but understanding the pathologies of the prenatal conditions and how to optimize the actual therapeutic interventions like antibiotic therapy might open a more promising window for improving the relevant outcomes and to focus the strive for novel effective therapies. The results thereby have implications for many disease entities far beyond the neonatal period and to focus research efforts on the detection of the early disease origins and the earliest possible intervention.

## Supplementary Information

Additional File 1: Additional Figures S1 – S3. Additional Figure S1. - Differences in number of 16S rRNA gene copies and microbial abundance and community composition between blank controls and amniotic fluid samples. Additional Figure S2. - Rarefaction curve for amniotic fluid samples. Additional Figure S3. - Bacterial load quantified via 16S rRNA gene-based qPCR.

Additional File 2: Additional Tables S1 – S3. Additional Table S1. - List of Amplicon sequence variants (ASV) detected in blanks. Additional Table S2. - Amplicon sequence variants (ASV) counts per sample before normalization. Additional Table S3. - Logistic regression with birth weight.

## Data Availability

All sequence data of the study are deposited in the short read archive of NCBI and accession number PRJNA1260988.

## Abbreviations

AF: Amniotic fluid
ASV: Amplicon sequence variant
BPD: Bronchopulmonary dysplasia
IVH: Intraventricular haemorrhage
LOI: Late-onset infection
LPS: Lipopolysaccharide
MDI: Mental developmental index
NEC: Necrotizing enterocolitis
NICU: Neonatal intensive care unit
PDI: Psychomotor developmental index
ROP: Retinopathy of prematurity
SGA: Small for gestational age
VPT: Very preterm
