# Supplementary material for: Association of specific microbiota taxa in the amniotic fluid at birth with severe acute and longer-term outcomes of very preterm infants: a prospective observational study
Source: BMC Med. 2025 Jul 18;23:431. doi: 10.1186/s12916-025-04259-9 (PMC12275360; doi:10.1186/s12916-025-04259-9)
Supplement: Supplementary file 1 — Additional File 1: Additional Figures S1 – S3. Additional Figure S1. - Differences in number of 16S rRNA gene copies and microbial abundance and community composition between blank controls and amniotic fluid samples. Additional Figure S2. - Rarefaction curve for amniotic fluid samples. Additional Figure S3. - Bacterial load quantified via 16S rRNA gene-based qPCR. [file 12916_2025_4259_MOESM1_ESM.pdf]

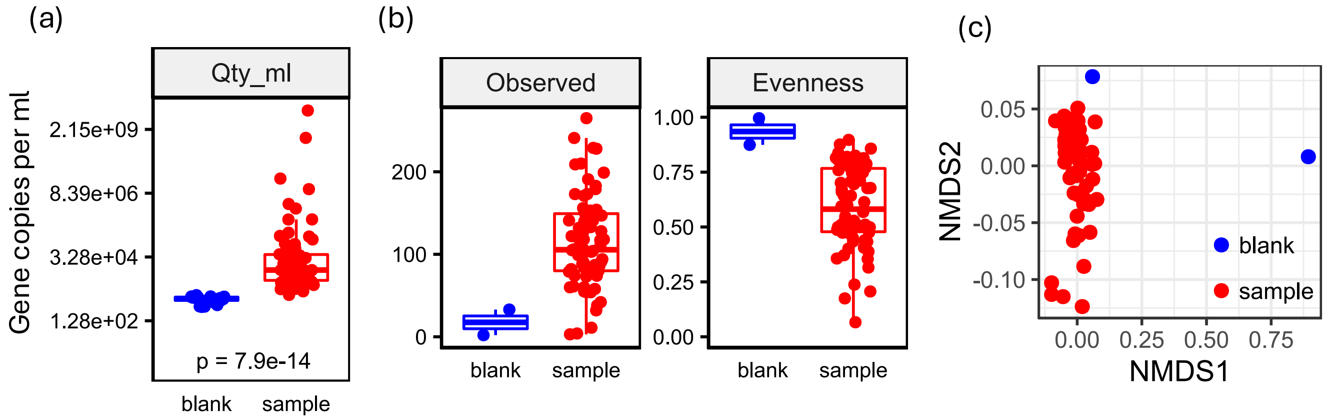

Additional Figure S1. Differences in number of 16S rRNA gene copies and microbial abundance and community composition between blank controls and amniotic fluid samples.

(a) Number of 16S rRNA gene copies quantified via qPCR were significantly lower in blanks compared to amniotic fluid (AF) samples.

(b) Alpha diversity (measured as number of observed ASV and evenness) confirmed lower diversity in blank controls with a significance of < 0.05.

(c) Beta diversity (NMDS plot of weighted Unifrac distances) differed between blank controls and AF samples.

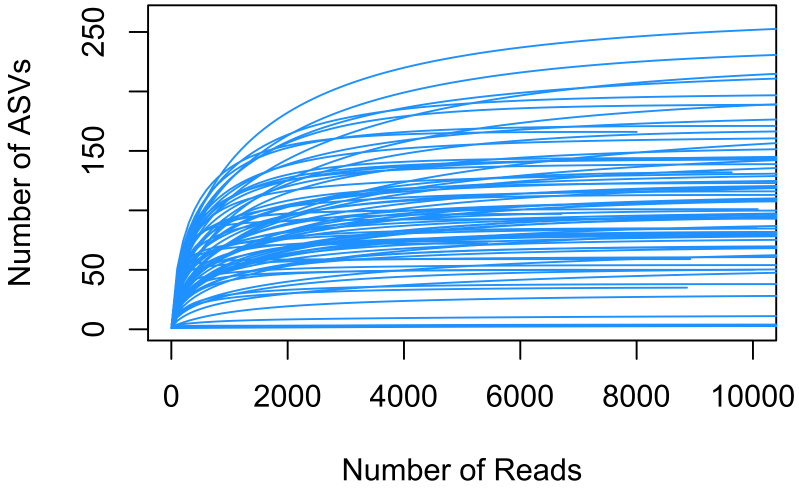

Additional Figure S2. Rarefaction curve for amniotic fluid samples.
Rarefaction curve showed sufficient sampling depth at 5434 reads.

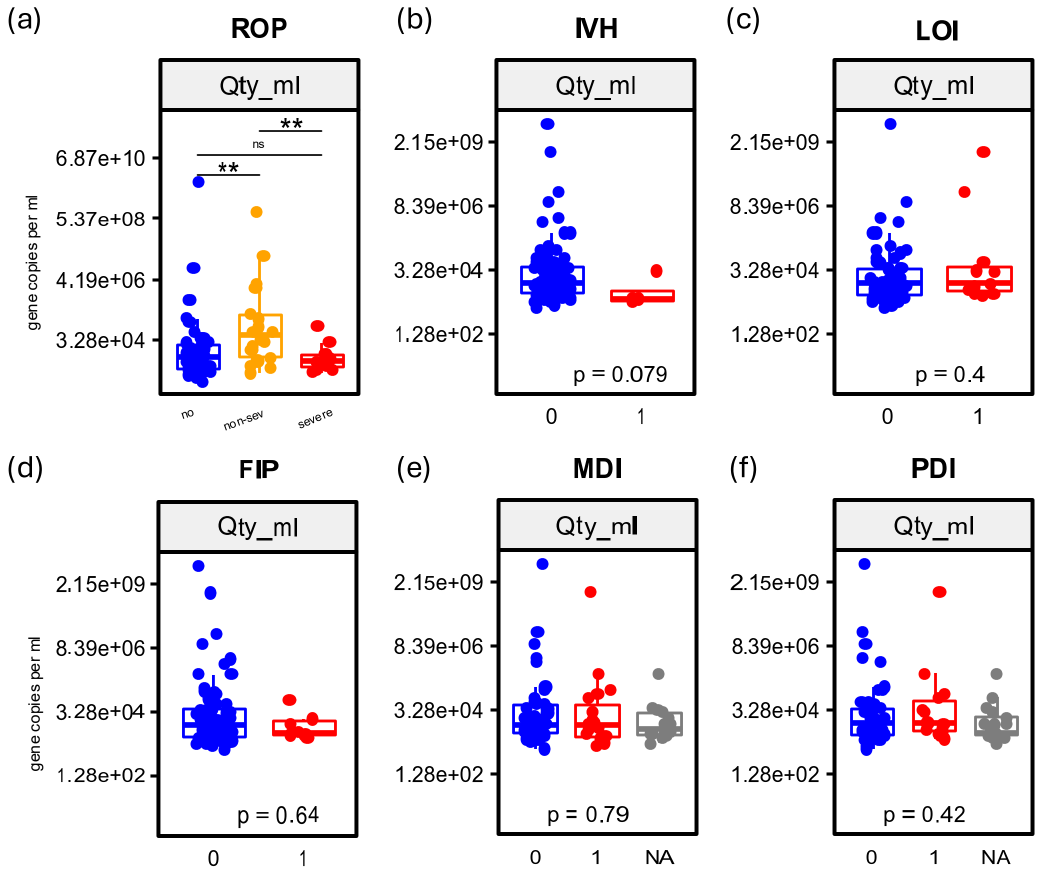

Additional Figure S3. Bacterial load quantified via 16S rRNA gene-based qPCR.
Number of 16S rRNA gene copies are displayed for the outcomes of ROP (a), IVH (b), LOI (c), FIP (d), MDI (e) and PDI (f). No significant differences were detected for IVH, LOI, FIP, MDI and PDI. For ROP, bacterial load was significantly higher in the non-severe ROP group. ROP, retinopathy of prematurity; IVH, intraventricular haemorrhage; LOI, late onset infection (>72 hours after birth); FIP, focal intestinal perforation; MDI, mental developmental index; PDI, psychomotor developmental index.
